# Optimizing a Novel Smartphone App to Prevent Postpartum Depression Adapted From an Evidence-Based Cognitive Behavioral Therapy Program: Qualitative Study

**DOI:** 10.2196/63143

**Published:** 2024-12-09

**Authors:** Adam K Lewkowitz, Melissa Guillen, Katrina Ursino, Rackeem Baker, Liana Lum, Cynthia L Battle, Crystal Ware, Nina K Ayala, Melissa Clark, Megan L Ranney, Emily S Miller, Kate M Guthrie

**Affiliations:** 1 Department of Obstetrics and Gynecology, Warren Alpert Medical School at Brown University, 222 Richmond Street, Providence, RI, United States, 1 4012741122; 2 Center for Digital Health, Brown School of Public Health, Providence, RI, United States; 3 Department of Psychiatry and Human Behavior, Warren Alpert Medical School at Brown University, Providence, RI, United States; 4 Department of Health Services Policy & Practice, Brown School of Public Health, Providence, RI, United States; 5 Yale School of Public Health, New Haven, CT, United States

**Keywords:** cognitive behavioral therapy, mothers and babies program, digital health, postpartum depression, smartphone application, FRAME for intervention adaptation, Framework for Modification and Adaptation, behavioral therapy, mental health apps

## Abstract

**Background:**

Low-income pregnant patients are at high risk of postpartum depression (PPD). Mothers and Babies (MB) is a cognitive behavioral therapy–based program that prevents up to 50% of de novo PPD when provided in person to low-income Spanish- and English-speaking people who are pregnant without depression. MB is limited by the need for trained personnel to support it. Transforming MB into a smartphone app may mitigate this key barrier.

**Objective:**

We aimed to use qualitative data from target end users to create and optimize MBapp, a novel app centered on the MB program.

**Methods:**

Draft wireframes of MBapp were created in English and Spanish with cognitive behavioral therapy–based modules adapted from MB. These wireframes included several features shown previously to sustain app engagement: (1) push notifications delivered at participant-preferred times; (2) text-, graphic-, and video-based content; and (3) gamification with digital rewards for app engagement. English- or Spanish-speaking individuals with public health insurance who were between 32 weeks gestation and 6 months post partum and owned smartphones were eligible to consent for individual in-depth interviews. Individuals with prior or current depression were excluded. Interviews were recorded, transcribed, and analyzed using deductive and inductive codes to characterize opinions about MBapp and perceptions of challenges and facilitators of use of MBapp or other perinatal or mental health apps. End user feedback led to major modifications to the wireframes. Each of these changes was categorized according to the FRAME (Framework for Modification and Adaptation), an established method of systematically reporting adaptations and modifications to evidence-based interventions via end user feedback. Recruitment ceased with content saturation, defined as 3 successive participants providing only positive feedback on MBapp’s wireframe, without further suggestions for improvement.

**Results:**

A total of 25 interviews were completed. Participants were racially and ethnically diverse, generally representing our target end user population, and 48% (n=12) of interviews were conducted in Spanish. Participants’ suggestions to improve MBapp were categorized within the FRAME as adaptations that improved either content or context to optimize reach, retention, engagement, and fit for end users. Specifically, the following features were added to MBapp secondary to end user feedback: (1) audio narration; (2) “ask a clinician” nonurgent questions; (3) on-demand module summaries accessible upon module completion; and (4) choice to defer assessments and start the next module. Participants also provided insights into features of perinatal or mental health apps they found appealing or unappealing to understand preferences, challenges, and negotiables or nonnegotiables for MBapp.

**Conclusions:**

Adapting MBapp to incorporate end users’ perspectives optimized our digital PPD prevention intervention, ideally increasing its appeal to future users. Our team’s next steps will confirm that MBapp is a feasible, acceptable intervention among English- and Spanish-speaking perinatal people at risk of PPD.

## Introduction

Approximately 15% of all pregnant women in the United States develop postpartum depression (PPD) [1-6], and more than 95% of people with PPD have persistent symptoms [7]. The reasons for such a high burden of persistent symptomatic burden from PPD are complex but include the following barriers: (1) obstetric providers who are not trained to care for psychiatric conditions and who do not routinely screen and treat for mental health conditions such as PPD; (2) a shortage of trained mental health providers to care for those who screen positive; and (3) stigma of receiving in-person mental health care, which prevents those with PPD from receiving effective treatment [8-14].

Thus, preventing PPD from occurring is crucial, particularly among individuals with unmet social needs such as financial stress or food insecurity that place them at higher risk for this condition than those without unmet social needs [1-6]. The US Preventive Services Task Force recommends that women at risk for PPD participate in 1 of 2 evidence-based psychotherapy programs shown to effectively prevent PPD from developing [15,16]. One of these programs is an in-person cognitive behavioral therapy (CBT)–based program, Mothers and Babies (MB) [17-19]. MB was created to prevent PPD among low-income English- or Spanish-speaking pregnant women and has been shown to be effective in diverse communities of low-income perinatal patients [17-19]. Though MB has been adapted to an asynchronous online intervention [18,20], access to the program generally remains limited to people who receive prenatal care in clinical settings affiliated with MB-trained providers, reducing MB’s dissemination potential.

App-provided mental health care has dramatically expanded access to mental health services through their ability to be scaled without reducing fidelity and their ability to reduce barriers and potentially stigma related to receiving in-person mental health care [8,21]. In addition, app-provided mental health care has been shown to effectively treat depressive symptoms and is commonly used in pregnancy (the majority of people who are pregnant download apps regardless of income) [22-24]. From a perinatal patient perspective, engaging with an app to improve somatic and mental health symptoms has become commonplace [25,26]. However, most mental health apps do not contain evidence-based psychotherapy [27,28], and, of the apps that do contain CBT elements, most do not contain content that has been specifically created for peripartum people [29]. For these reasons, we aimed to adapt the evidence-based MB course, ultimately creating a 9-module, app-based intervention, entitled MBapp, from the original 6-session in-person group intervention. In this paper, we present findings from individual in-depth interviews with pregnant or postpartum individuals regarding optimal perinatal or mental health apps. We also use an established method of systematic reporting of adaptations and modifications to evidence-based interventions via end user feedback to describe how incorporating end users’ suggestions optimized MBapp for future pregnant or postpartum users. To our knowledge, this study represents the first to systematically report the process of adapting an existing in-person curriculum to a perinatal mental health app before the intervention was deployed.

## Methods

### Creating MBapp Draft Wireframe

MB materials are publicly available [30]. Once downloaded, MB’s curriculum for course leaders and the participant workbook served as the starting point for MBapp. The draft wireframe was created by this study’s principal investigator (AKL), who was supported by his coauthors—a multidisciplinary team with expertise in perinatal mental health, digital health interventions, and qualitative research methods. To create the draft wireframe, MB’s curriculum was first adapted to be delivered asynchronously on a smartphone app. Prior digital adaptations of MB reported that end users requested additional alterations to the digital draft content by further simplifying language and adding more visuals [20,31,32]. Thus, in MBapp’s initial draft wireframe, paragraphs of text or worksheets in MB were re-restructured to include short, direct phrases, colorful graphics (some from the MB curriculum), and pictures of pregnant and postpartum people and young infants. Some of MB’s videos were included within MBapp to allow users to learn the curriculum via a combination of text, graphics, and videos. The draft wireframe also contained ecological momentary assessments and gamification, features that have been shown in other perinatal mental health apps to increase the likelihood of sustained user engagement [33-35]. Specifically, MBapp included daily push notifications at users’ preferred times that prompt them to complete a mood scale. Their scores on this scale send them to pertinent content within the app. In terms of gamification, responding to at least 3 of the 7 daily ecological momentary assessment requests to complete the mood scale generated a digital reward, which can be visualized in a digital trophy case. Original MB creators confirmed the draft wireframe was adherent to CBT principles and retained fidelity to MB. Once the draft wireframe was completed, qualitative feedback was obtained from target end users to further adapt the intervention.

### Study Population

Participants were recruited from among those receiving perinatal care at a clinic that exclusively serves patients with government pregnancy-related health insurance (Medicaid), as having perinatal Medicaid insurance is only available for low-income patients who are pregnant in our state (Rhode Island, United States) [36]. To be eligible for this study, participants were English- or Spanish-speaking, aged at least 18 years, owned a smartphone, and were either in the third trimester of pregnancy or had an infant that was born within the prior 6 months. As MB prevents—but does not treat—PPD, volunteers were excluded if they reported prepregnancy or current anxiety or depression, an active (or within last 5 years) prescription for psychotropic medications, active engagement in psychotherapy, untreated substance use disorder, or if they were unable to consent. Potential participants were approached in person during routine antenatal or postpartum care visits. Those interested, who remained eligible after screening, provided written informed consent in person before the interview was scheduled. Participants who did not complete their interview after 3 no-shows at interview appointments were not rescheduled.

### Data Collection

Individual in-depth interviews were all conducted via video teleconferencing at the participant’s preferred schedule and in their preferred language (English or Spanish). Each interview was conducted by the same investigator (AKL), who followed a semistructured interview agenda that divided the interview into two components. First, the interview discussed the participant’s personal experiences, preferences, and challenges with apps, particularly those that focus on perinatal or mental health. Then, the investigator shared his computer screen to review distinct components of MBapp’s wireframe with the participant and obtain page-by-page feedback. All interviews were audio-recorded, transcribed by a professional transcription service, and reviewed for accuracy with identifiers redacted. Participants were asked their race and ethnicity.

### Using an Evidence-Based Framework to Determine Sample Size

Similar to other web-based digital adaptations of MB [18,20], a human-centered design approach was used to optimize MBapp based on end user feedback about the intervention itself and other, optimal perinatal or mental health apps. We recorded MBapp’s evolution according to the FRAME (Framework for Modification and Adaptation) [37], an established method of systematic reporting adaptations and modifications to evidence-based interventions via end user feedback. The FRAME provides insight about when MBapp’s wireframe was modified during the development process and the reason for the modification. Feedback on MBapp’s themes, content, and function were extracted from the data and, after 5 to 7 interviews, the draft wireframes were revised to incorporate this feedback. The new iteration of the wireframe was then presented to subsequent participants to allow revisions suggested by earlier participants to be seen and commented on by subsequent participants. We anticipated recruiting up to 36 participants and planned to cease recruitment with content saturation, defined as 3 successive participants providing only positive feedback on MBapp’s wireframe, without further suggestions for improvement.

### Data Analysis

Authors AKL, MG, and KMG generated a preliminary codebook for the interviews based on the semistructured interview agenda. This initial version of the codebook was limited to deductive codes. Authors AKL, MG, KU, RB, and LL then collaborated via consensus coding in stages. First, two transcripts were coded collaboratively, and additional codes were added inductively to the codebook. Some of these codes were added de novo whereas other inductive codes were based on deductive codes that were reorganized to better capture data. Second, each of these authors independently coded a third transcript, and the authors met as a group to reconcile coding discrepancies. In this manner, consensus coding was achieved and a codebook comprising deductive and inductive codes was finalized. Then, all transcripts—including those that had already been reviewed—were coded independently by two authors (AKL and either KU, RB, or LL). A third coauthor reviewed transcripts and codes when coding disagreements could not be resolved (MG). All coding was then inputted into NVivo (Lumivero), and coded excerpts were organized and synthesized into thematic memos and discussed by the coding team. This study was reviewed and approved by Women & Infant’s Hospital of Rhode Island’s institutional review board prior to the consenting of the first participant.

### Ethical Considerations

This study was approved by the institutional review board of Women & Infants Hospital of Rhode Island (submission WIH 21-0021; approved on September 7, 2021). All participants were assigned a study identification number, which was stored on a password protected file and was only utilized to coordinate the $50 electronic gift card, which was provided to each participant after the study interview was completed. Otherwise, all data generated during the interview was deidentified and stored in a password protected computer network.

## Results

### Characteristics of Study Participants

From August 2022 to August 2023, a total of 33 patients were consented, and 25 individual in-depth interviews were conducted. There were no differences in language of scheduled interview, patient-reported race, or parity between those who consented and did versus did not complete the interview. The sociodemographic and obstetric characteristics of participants who completed their interviews are described in Table 1. In brief, 24% of participants were aged 35 years or older, 24% self-reported as White, and nearly half (n=12, 48%) of interviews were conducted in Spanish. Approximately the same proportion of participants were pregnant versus post partum, and over half of participants were first-time parents (ie, either about to deliver or had recently birthed their first child).

**Table 1.** Sociodemographic and obstetric characteristics of study participants (N=25).

Characteristic	Value
Age (years)	
Median (IQR)	29 (24-35)
Minimum, Maximum	20, 43
Advanced maternal age (35 years or older at time of delivery), n (%)	6 (24)
Race, n (%)	
American Indian or Alaska Native	3 (12)
Asian	2 (8)
Black	5 (20)
White	6 (24)
Other^a^	9 (36)
Ethnicity, n (%)	
Hispanic	13 (52)
Language of interview^b^, n (%)	
English	13 (52)
Spanish	12 (48)
Primiparous (pregnant with first child or recently delivered first child), n (%)	13 (52)
Pregnancy status, n (%)	
Pregnant	13 (52)
Postpartum	12 (48)

a Other: multiracial (White and Black): 4; multiracial (White and American Indian or Alaska Native): 3; multiracial (Black and American Indian or Alaska Native): 2.

b Four participants spoke a combination of English and Spanish. Their interview language was categorized as the language that most of the interview was conducted in.

### Perspectives on Optimal Features Within Perinatal or Mental Health Apps

Twenty-one of the 25 participants reported using at least one pregnancy-related app, and 2 of the 25 participants accessed a mental health related app during the perinatal period. The general consensus among perinatal app users was they were more likely to engage with an app if it provided pertinent, updated, and easy-to-understand text and video education either via a push notification or an email and contained digital rewards or games. Many perinatal app users noted that they turned to apps to receive perinatal education between prenatal care appointments or to supplement perinatal education received during these appointments:

The doctor focuses on talking about the pregnancy, focuses on the baby or they just look at your vagina and then [participant waves arms to mimic dismissing someone, as if doctor had instructed her to leave the room]. It was not much about me…[My app] helped me learn about what I wanted to know

[Postpartum, English-speaking, first-time mother]

Another consistent theme that emerged was that many participants engaged with perinatal apps to learn from people similar to them (those who were pregnant or had recently given birth):

[After my baby was born] they would call me, maybe it was a doctor or nurse, and they would ask me questions, but it’s not the same. I can’t explain it. It’s not the same as seeing a mom that is the same as me [who] is saying, “Look, do this. No, do this. Look,” and they show me with their own baby.

[Pregnant, Spanish-speaking mother with prior children]

Though apps were consistently described as useful throughout the perinatal period, the majority of users who had delivered at least one child at the time of the interview noted that apps were particularly helpful after delivery. The need for perinatal apps in the postpartum period was described in language suggesting that apps help fill the gap between in-person medical care from birth until 6 weeks after delivery. The long interval between medical appointments left participants feeling unsupported by their prenatal care team, and apps helped to provide support:

[When I left the hospital] they did give me a pamplet that had some…breastfeeding classes and stuff like that [but] I lost the paper…Now I have questions [on breastfeeding] and don’t have anywhere to go, but wait for the next doctor appointment… [My app] gives an idea of how much milk I should give him. Super helpful.

[Postpartum, English-speaking first-time mother]

While prenatal apps were often used for additional perinatal education, there was a recurrent theme that these apps—such as prenatal care—focus more on the baby than on the mother:

Apps for mother and baby focus more on the baby than the mother…[just like in real life]. An example, when the mother gives birth to the baby, everyone is concerned about the baby. Hardly anybody worries about the mother, but the mothers go through a difficult process.

[Pregnant, Spanish-speaking mother with prior children]

Participants in this sample were much less likely to use mental health apps than perinatal apps. Mostly, participants attributed this difference to a lack of knowledge that such resources were available, not due to lack of interest in perinatal mental health apps. For example, one participant shared that she had depressive symptoms after delivery and informed her midwife of these symptoms at her postpartum visit:

She told me it was normal. She [gave] me a sheet of paper to…see if I had suicidal thoughts, if the depression was severe…and things like that. [Then] she told me to find a counselor. That I needed one. I started to look for a counselor…I wish she had told me about this [mental health apps]. I would have used one and found it useful.

[Postpartum, Spanish-speaking first-time mother]

Once the interview shifted toward talking about how MBapp aimed to provide app-based mental health support for perinatal people, the majority of participants responded very favorably, even before viewing any of MBapp’s wireframe:

I feel like an app that talks about mental health and they make it fun with the game and the video and the colors and everything, I think it could be useful. I would’ve liked something like that—or even now, I would like it.

[Pregnant, English-speaking first-time mother]

### Adapting the MBapp Wireframe

The MBapp wireframe was adapted in multiple ways through end user feedback. Participants were asked about specific features in perinatal or mental health apps, and whether each feature was appealing (or not) and useful (or not). In addition, each participant viewed at least two distinct sections of MBapp’s wireframe and provided direct feedback on the appearance, appeal, content, and usability of each page. This feedback led to minor changes in MBapp’s font size or color scheme. Each participant also was asked general questions on how MBapp should be modified to encourage initial and sustained user engagement from the third trimester until 6 months post partum. All feedback from participants was considered by the study team and adaptations were incorporated into MBapp’s wireframe. Table 2 highlights the major adaptations made to MBapp’s wireframe during qualitative interviews and categorizes these changes according to the FRAME [37]. Of note, participant feedback resulted in both significant and nuanced changes to MBapp but did not alter MBapp’s core CBT content; thus, these adaptations did not affect MBapp’s fidelity to the evidence-based MB curriculum.

MBapp’s wireframe was originally created to mirror MB’s programmatic structure, in which a module on parenting education and infant care occurred midway through the curriculum and participants were instructed to complete daily mood scales between each in-person session, regardless of content. Based on end user feedback, MBapp was restructured such that the parenting module was first. We also edited the wireframe to provide users with the option to proceed with the daily mood scales for one week before accessing the next module or to defer these assessments in lieu of moving on immediately to the next module.

**Figure 1.**
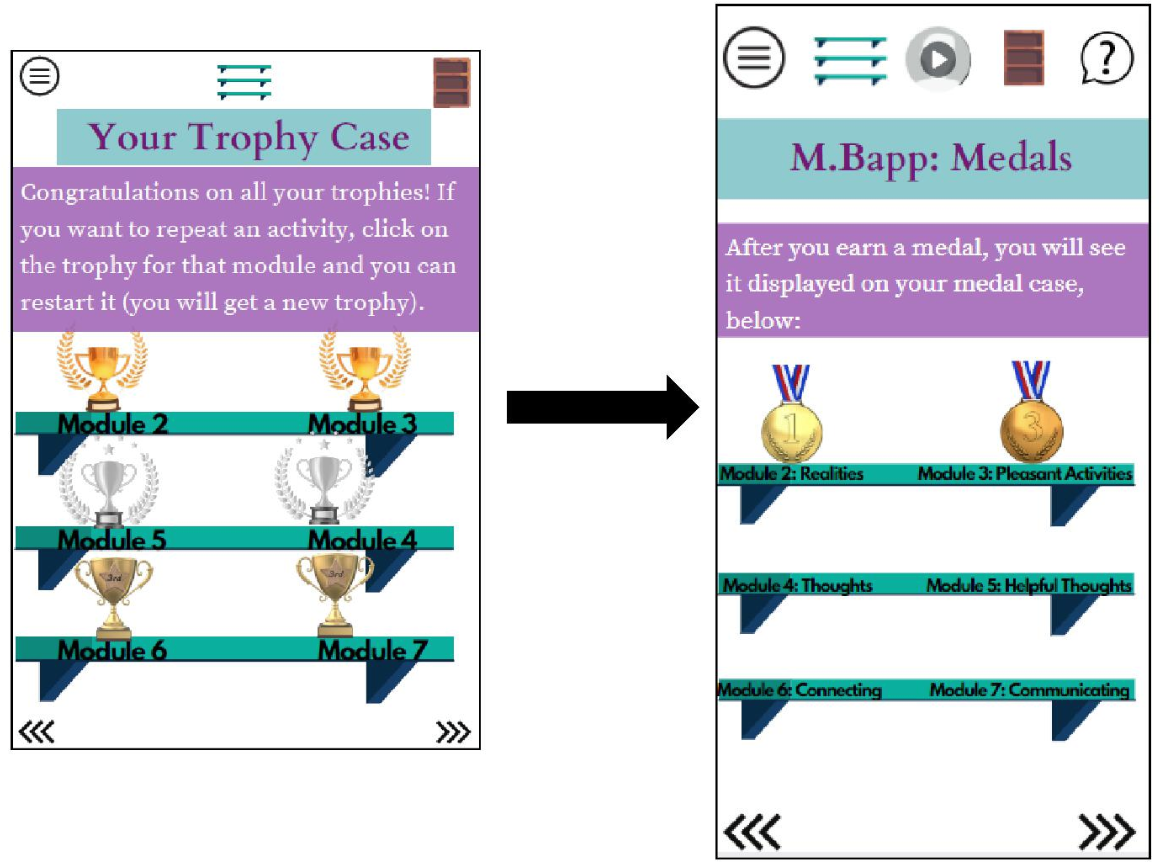
Visual representation of major adaptations to MBapp’s draft wireframe (left) and final wireframe (right), using the digital trophy case as an example. In MBapp’s draft wireframe (left), the graphic was smaller; the digital rewards were trophies; and the icons in the header (which appear on every page) were limited to the table of contents, the digital reward icon, and the bookshelf (from left to right). Also, modules 4 and 5 were mislabeled. The final wireframe (right) represents adaptations to MBapp due to end user feedback, which included larger graphic size, medals as digital rewards, more text description for each module, less text description on the page itself (the text is spread over to two pages), and additional icons in the header (audio feature is in the middle, and the question mark icon is how users ask a provider a nonurgent question). The updated header appears on every page.

Participants recommended adding multiple new features to increase the likelihood of sustained engagement. These included modest financial reimbursement tied to responding to the daily mood scale and the option for audio narration, as well as an “ask a clinician” feature in which users can ask a nonurgent question to a member of the MBapp clinical team, who would respond through the platform. End users also desired short module summaries to be added after each module so they could review high-yield content without repeating the entire module. Module summaries now appear automatically on a digital bookshelf upon completion of each module. Figure 1 provides an example of how MBapp’s wireframe was adapted from the initial to final versions.

**Table 2.** Major end user–requested adaptations to MBapp’s wireframe, categorized according to the FRAME^a^ [37].

MB^b^ feature	MBapp adaptation	Classification^c^	Reasons^c^	Nature of the content modification^c^	Timing of change implementation	Participant recommendation addressed
Not in MB	“Ask a clinician” nonurgent questions	Content: adding elements	Recipient: access to resources	Improve fit with recipients	After interview #16	Resource for perinatal or mental health advice, support, or education
Not in MB	Audio narration, to be turned on or off on each page	Context:adding elements	Recipient: first or spoken languages	Improve fit with recipients	After interview #11	Increase engagement with MBapp via additional method to deliver content
Mood assessments given between in-person classes	Choice to defer week of mood assessments and pass immediately to next module	Context: format	Recipient: cognitive capacity	Increase retention	After interview #11	Flexible schedule
Parenting education module in middle of curriculum	MBapp module order restructuring, with parenting module in the beginning	Content: reordering of intervention modules or segments	Recipient: cognitive capacity	Improve fit with recipients	After interview #6	Optimize MBapp flow by removing parenting education from middle of curriculum
Not in MB	Module summaries to access high-yield content after module is completed	Content: adding elements	Recipient: cognitive capacity	Increase retention	After interview #6	Practical, high-yield content for fast review
Not in MB	Small financial reimbursement based on number of responses to ecological momentary assessment prompts	Content:tailoring, tweaking, and refining	Recipient: motivation and readiness	Increase reach or engagement	After interview #21	Increase meaningful engagement with MBapp via gamification

a FRAME: Framework for Reporting Adaptations and Modifications.

b MB: Mothers and Babies.

c As defined by FRAME [37].

## Discussion

### Principal Findings

To our knowledge, this study represents the first to systematically report the process of adapting an in-person curriculum to a perinatal mental health app before the intervention was deployed. Indeed, this paper presents qualitative data from 25 diverse pregnant or postpartum perinatal patients with Medicaid insurance, describing their perspectives on perinatal or mental health apps and design recommendations for MBapp, a novel app based on an in-person CBT program shown to effectively prevent PPD among low-income patients [15,16]. The majority of participants used perinatal apps to receive on-demand pregnancy or postpartum education, and many preferred learning from others with similar experiences. Comparatively, mental health app use was less common, but this lack of uptake was attributed to lack of awareness, not disinterest.

Participants’ perspectives and recommendations were incorporated into MBapp’s wireframe, which led to minor adjustments in MBapp’s aesthetics and major adaptations to MBapp’s structure and features. All major adaptations were categorized according to the FRAME [37]. As each modification was derived directly from qualitative feedback from individual end users, incorporating minor and major adaptations to MBapp’s appearance, structure, and features should increase its appeal to future users.

### Comparison With Prior Work

MB was originally created as 6 in-person 2-hour group sessions [17] and has been implemented successfully in nontrial, community settings [38]. Since its inception, MB has been adapted into multiple formats. For example, 1 in-person MB curriculum comprises 10 individual sessions occurring during prenatal visits or at home [19] and can include supplemental text messages to participants [32]. There are also two asynchronous, online adaptations of MB, one for perinatal women and one for pregnant adolescents at risk for PPD [20]. Each digital adaptation of MB (text-based or online) included mixed methods research to optimize the intervention according to end user perspectives, and this feedback led to simplified language and more visuals in the final digital MB intervention [20,31,32]. Based on prior investigators’ experience with adapting MB to a digital intervention, we assumed that adapting MBapp would also need to include simplified and direct language and visual engagement via graphics and pictures of perinatal individuals and infants. The initial wireframe was developed with these features. Thus, all participants could provide granular feedback on features that were included or should be added to MBapp to encourage engagement, instead of focusing on giving the investigative team feedback on reducing the amount of text or adding visual appeal to the wireframe. It is important to note that only the online MB program for high-risk perinatal adolescents used formative qualitative interviews to adapt MB via a structured framework such as the FRAME, as is described in this paper. Incorporating this evidence-based approach ensured that all major MBapp modifications were well-justified and provided transparency to inform how future data on MBapp’s effectiveness may be interpreted.

### Strengths and Limitations

Our study had many strengths. Each aspect of intervention creation and adaptation was grounded in evidence-based practices: (1) MBapp’s core content was derived from an in-person CBT curriculum known to be highly effective for PPD prevention [17-19]; (2) the individual in-depth interviews were performed according to established and recommended qualitative research practices as described by coauthors of our paper [39-41]; and (3) MBapp was adapted using a standard, commonly used framework for modifying established interventions [37]. In addition, our participants were diverse in terms of race, ethnicity, language, and parity, helping to ensure that different perspectives and opinions were included in our digital perinatal mental health intervention. Lastly, MBapp was created with a multidisciplinary team with expertise in perinatal mental health, digital health, and qualitative research, ensuring the digital health intervention was created via best practices.

Nevertheless, our study is not without limitations. First, our study population was comprised of 25 participants. Though a standard sample for a qualitative research study, this study population cannot possibly represent all opinions and perspectives of perinatal patients. Second, recruitment was limited to one obstetric clinic, which may reduce transferability of findings to individuals within different types of clinics (eg, academic vs community-based obstetric clinics). Third, MBapp will be a fully functional native app available on iTunes (Apple Inc) and Google Play (Google LLC). Compared to a web-based intervention that can be easily and cheaply modified after creation, native apps such as MBapp incur significant software developer costs for any modifications postcoding. Though we cannot alter MBapp postproduction as has been carried out in web-based perinatal mental health interventions [42], we are optimistic that using the FRAME and end user feedback has optimized MBapp’s wireframe to the point that additional edits are unnecessary. Lastly, though MBapp was deemed to have preserved fidelity to MB’s curriculum by MB’s original creators, it remains an empirical question whether an asynchronous digital education delivered via smartphone app is comparable to a 12-hour in-person group education led by a trained facilitator. Thus, the effect of MBapp on PPD will need to be explicitly and intentionally tested in future randomized trials.

### Conclusions

In conclusion, the majority of our participants—pregnant or postpartum individuals with public health insurance—used apps to obtain on-demand perinatal education, and this expertise was leveraged to update and enhance a novel smartphone adaptation of MB. MBapp’s wireframe was tailored to the unique needs of app-based interventions and to feedback from target end users, which may result in sustained user engagement once the app is deployed. Our team’s next steps will be to confirm that MBapp is a feasible, acceptable intervention among English- or Spanish-speaking pregnant and postpartum people at risk of PPD through a pilot randomized trial. Ultimately, we aim to demonstrate that MBapp is an effective, scalable intervention that can prevent PPD on a population level.

## Abbreviations

CBT: cognitive behavioral therapy
FRAME: Framework for Modification and Adaptation
MB: Mothers and Babies
PPD: postpartum depression
